# Deep Learning Network for Multiuser Detection in Satellite Mobile Communication System

**DOI:** 10.1155/2019/8613639

**Published:** 2019-03-04

**Authors:** Guan Qing yang, Wu Shuang, He Ya-Ru

**Affiliations:** ^1^College of Engineering, Xi'an International University, Xi'an 710077, China; ^2^College of Electronic and Information Engineering, Shenyang Aerospace University, Shenyang 110136, China

## Abstract

A multiuser detection (MUD) algorithm based on deep learning network is proposed for the satellite mobile communication system. Due to relative motion between the satellite and users, multiple access interference (MUI) introduced by multipath fading channel reduces system performance. The proposed MUD algorithm based on deep learning network firstly establishes the CINR optimal loss function according to the multiuser access mode and then obtains the best multiuser detection weight through the steepest gradient iteration. Multilayer nonlinear learning obtains interference cancellation sharing weights to achieve maximum signal-to-noise ratio through gradient iteration, which is superior than the traditional serial interference cancellation algorithm and parallel interference cancellation algorithm. Then, the weights with multiuser detection through multilayer network forward learning iteration are obtained with traditional multiuser detecting quality characteristics. The proposed multiuser access detection based on deep learning network algorithm improves the MUD accuracy and reduces the number of traditional multiusers. The performance of the satellite multifading uplink system shows that the proposed deep learning network can provide high precision and better iteration times.

## 1. Introduction

Due to high-speed relative motion between mobile users and satellites in the satellite mobile communication system, different users access with the satellite at different elevation angles and multipath channel between satellite and user links is fading. These factors are creating obstacles for multiuser detection. In the case of limited bandwidth system, multiuser access detection (MUD) is an important issue in satellite mobile communication systems.

In the early literature, Cao and Viswanathan [1] proposed a method based on transformation of training sequences for single-user detection; the performance based on transforming training sequences is poor because the algorithm needs to add redundant information to make the signal transmission efficient. In the literature [2, 3], a soft iterative method was proposed for multiuser signal detection, but soft iteration required too much user information and it was not easy to achieve convergence.

Since multiusers accessed the satellite system at different elevation angles, different access carrier frequency offsets (CFOs) introduced multiuser access interference (MUI), so it was difficult to implement single-tap FDE to achieve multiuser detection. Some related research studies had been designed for multiuser detection. Tang and Heath [4] proposed a joint MUD scheme for MIMO. On this basis, in [5], CP was used to perform multiuser detection by accurately estimating the frequency offset. Zhang and Gao [6] proposed a blind scheme for multiuser uplink with large antennas. However, literatures [4–6] were proposed based on CP, and their solutions relied on searching for sampling. In this case, it suffered significant performance degradation, especially in the case of relatively large delay spread.

Blind user detection did not require a priori information, which effectively improves transmission efficiency. Therefore, Zhang and Gao [6] proposed a blind detection algorithm. At present, based on this, in [7, 8], a combination of advanced technologies, such as space-time processing and interference cancellation, which improved transmission performance of the system, is proposed. Karakaya et al. [9] proposed an improved Kalman filter (KF) for multiuser detection, which requires a longer training sequence. Cao et al. [10] proposed LS-based algorithm (least squares) and MMSE (least mean square error) for multiuser interference, but they are not accurate. Chang et al. [11] proposed a multiuser detecting method, which needs cancel interference caused by carrier frequency with a high degree of complexity. A new algorithm based on time domain carrier frequency offset compensation algorithm was proposed in [9] for multiuser detection, but its accuracy was low. Cui in [10] proposed the joint iterative detection algorithm, which required a lot of matrix transposition operations and high complexity.

In recent years, literatures researched on the compressed sensing reconstruction algorithm for multiuser detection. Abebe and Kang [12] proposed an iterative sorting least squares (IORLS) algorithm for detecting multiuser signals. Based on this, orthogonal signal tracking (OMP) was used in [13] to reconstruct the signal for fast multiuser signal detection. An iterative support detection (ISD) algorithm had been proposed in [14], and a structured iterative detection (SISD) algorithm was proposed in [15] to recover multiple sparse signals. In [16], an approximate message delivery mechanism was proposed to reconstruct the signal. On this basis, Wei et al. [17] had proposed the application of this message for multiuser detection. On this basis, Donoho et al. [18] introduced expectation-maximization (EM) into multiuser detection and was named “joint-EM-AMP algorithm.” This algorithm could achieve good bit error rate (BER) performance by jointly utilizing structured sparsity of prior information. Wang et al. [19] proposed a dynamic compression-aware algorithm for more practical scenarios.

For nonlinear transformation of single-layer network, the related literature also discussed and optimized in detail. For blind channel estimation of MIMO communication systems, AsadUllah et al. [20] proposed fuzzy logic-empowered opposite learning algorithm, which adopted mutant particle swarm optimization to obtain MMSE and BER performance. However, the single-layer nonlinear transformation of the algorithm reduces system performance.

Especially for the NOMA system, in literature [12, 21–23], a multiuser detection (MUD) algorithm was proposed. Since UEs of the NOMA system randomly transmitted data, they must perform blind activity detection. Wang and Yin [14] proposed compressed sensing (CS) algorithms. Wang et al. [24] proposed a modified version of the original ISD algorithm. Wang et al. [24] summarized multiuser detection method for the satellite mobile communication system. Literature [25] presents a joint ML-based CFO estimation method, but the complexity of this method was relatively high and was not suitable for satellite systems. In [26–28], the PIC algorithm for multiuser detection was proposed. Durand et al. [29] proposed the SIC algorithm based on weighting to detect multiuser in LTE-A systems, which is to improve the signal-to-interference ratio SINR. Kiayani et al. [30] proposed an improved PIC algorithm; the complexity was large since interference matrix transposition operation was very large, and the number of subcarriers was proportional.

For multilayer networks analysis, the related literature also discussed about CNN architecture. Yinghao et al. [31] proposed a multilayer neural network based on CNN for target recognition analysis, in order to deal with waste classification and obtain better resolution. Simulation results showed that the classification accuracy is higher than 90% under two different testing scenarios. Similarly, Albawi et al. [32] proposed a method for touch recognition, which was also implemented by CNN network. The proposed system outperformed other classification algorithms in terms of classification ratio. Simultaneously, the same CNN in [33] was also used in the Biomimetic Pattern Recognition to obtain a higher recognition rate. However, the above literatures all used the CNN network for visual feature recognition.

For the feature extraction of multilayer neural networks, Chao et al. [34] adopted a multilayer deep neural network with DBN architecture to acquire emotions through EEG signal acquisition. Compared with the CNN architecture, the DBN architecture was more suitable for processing transform domain features and achieving higher analysis accuracy. However, the algorithm was currently only for EEG signal analysis. Similarly, for multilayer neural network fusion decision, Wei et al. [35] proposed a weight-based fuzzy decision algorithm to achieve emotion recognition. The analysis was performed by multisource data decision fusion, which was including electroencephalography (EEG), electrocardiogram (ECG), respiration amplitude (RA), and galvanic skin response (GSR). However, the algorithm was used for data fusion direction.

## 2. System Model and Problem Formulation

### 2.1. Satellite-to-Ground Channel Model

If the shadow fading follows the Nakagami distribution, the Abdi star fading model was formed [36]. So, it is common for satellite-to-ground link model to use probability density functions, such as Rician model, Loo model, and Rician-Lognormal model. As a supplement to satellite-to-ground communication system, the satellite service scenario is mainly about wilderness area and open ground area, the shadow is less, and Clarke [37] also proved the correctness of this condition.

Some related literatures also have carried out studies on the satellite ground link channel model fitting through the measured data. Loo et al. [38], in view of the shadow effect on the signal propagation induced by trees, established the shadow effect model, but the application of the model has some limitations. Abdi et al. [39] established the urban road shadow (ERS) model for the satellite ground link with carrier frequency field between 870 MHz and 1500 MHz, and the model is suitable for the 1.5 GHz band channel fading distribution. In the literature [40], an integrated (CEFM) model is obtained by the integrated ERS model and the EFM model, which can be applied to a larger elevation range of 20 degrees to 80 degrees. Hess has used ATS-6 satellite to establish a small-scale and large-scale satellite ground link channel model within the city of 1200 km [41]. According to the ESA satellite ground link access elevation data, ERS model is put forward in literature [42], and the transmission channel model of terrestrial mobile satellites is given in [43], the establishment of *L*-band elevation model of this band access for multiple users.

**Figure 1:**
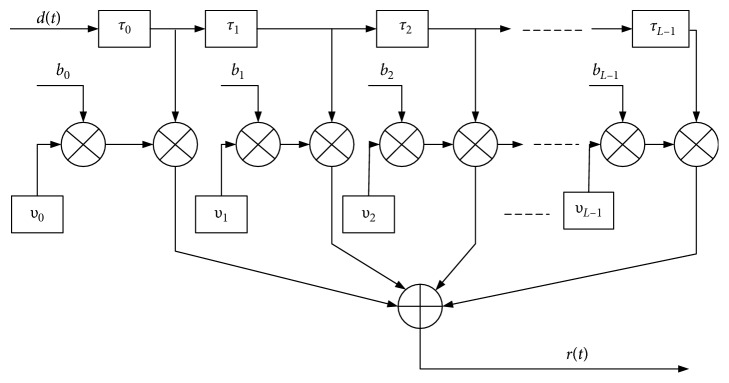
Channel model for satellite-to-ground link based on tapped delay line.

In this paper, the satellite-to-ground channel model is established using the measured results of the German Aerospace Research Center [42], and the multiuser access elevation model is established using the test data of the ESA (European Space Agency) in [37]. In this paper, the theory of tapped delay line channel model is used to establish the satellite-to-ground channel model.  Figure 1 shows frequency selective channel for satellite-to-ground link model based on tapped delay line.

The specific method is to simulate the signal amplitude fading through the tapped delay line filter. Firstly, it is assumed that the scattering body is divided into several clusters, and the bandwidth of the signal transmission bandwidth is not resolved within each cluster. Then, the multicluster is used to model the satellite-to-ground link.

In the tapped delay line model, each tap represents a set of a plurality of delay paths with the same sum, but also the time delay path changes due to different flat fading amplitudes.

The tapped delay line model for satellite-to-ground link, multipath channel impulse response is composed of different delay characteristics; the channel modeling method is established for satellite-to-ground channel model following the tapped delay line.

Define *h*(*t*, *τ*) multipath propagation delay and time channel impulse response function, for different *τ*'s; *h*(*t*, *τ*) is not related to each other. For determining the time delay *τ*, *h*(*t*, *τ*) is a stochastic process with mean complex Gauss time variation, and the impulse response *h*(*t*) is with the amplitude characteristics of flat fading. Therefore, the time-varying impulse of the multipath channel can be expressed as follows:

(1) $$\begin{array}{c} h\left(t,\tau \right)=\overset{L-1}{\underset{l=0}{\sum }}b_{l}\upsilon_{l}\left(t\right)\delta \left(\tau -\tau_{l}\right), \end{array}$$

where *τ*_*l*_ is the path of the transmission delay; *υ*_*l*_(*t*) is a complex Gauss process; *l* is expressed as the path delay component, and the fading of the path is induced by the Doppler power and power spectrum; *b*_*l*_ is expressed as the delay coefficient, the square root of the value for the average *l*th path delay power.

*υ*_*l*_(*t*) can be expressed in a delay interval at different incident angles' weighted path. The measured results with the German Aerospace Research Center are proposed in [36], which are about the rural environment, urban environment, suburban environment, with a signal carrier frequency of 1.82 GHz.

### 2.2. Multiuser Access Model

The Doppler shift caused by satellite motion is regular. At the same time, for defined mobile user, the Doppler shift is determined by the velocity of the high-speed motion and the elevation angle of the user. The Doppler shift introduced by high-speed satellite motion can be approximately equal.

Defining *x*(*n*) as the transmitted signal, *ξ* as the frequency offset factor, and *h*(*n*, *l*) as the impact response channel, the received *y*(*n*) can be expressed as

(2) $$\begin{array}{c} y\left(n\right)=\overset{L-1}{\underset{l=0}{\sum }}x\left(n\right)h\left(n,l\right). \end{array}$$

After introducing frequency offset interference, the received signal can be expressed as

(3) $$\begin{array}{c} y\left(n\right)=\overset{L-1}{\underset{l=0}{\sum }}x\left(n\right)h\left(n,l\right)\exp\left(\frac{j2\pi n\xi }{N}\right). \end{array}$$

The receiving end performs *N*-point DFT demodulation on the received band offset signal, and after serial conversion, we could obtain frequency domain signal, which can be expressed as

(4) $$\begin{array}{c} Y\left(k\right)=X\left(k\right)H\left(k\right)C\left(0\right)+\underset{l\neq k}{\overset{N-1}{\underset{l=0}{\sum }}}X\left(l\right)H\left(l\right)C\left(l-k\right), \end{array}$$

where *H*(*k*) is the channel frequency response and *C*(*k*) is the interference introduced in the frequency domain. The first term indicates the part without carrier interference. The frequency offset causes change for amplitude and the rotation caused by carrier *k*. The second term is the intercarrier interference caused by the remaining subcarriers of carrier *k*. As can be seen from the above formula, when *ξ*=0, *C*(0)=1. This means, when the carrier frequency offset is zero, the interference term coefficient is 1, which means no interference would occur. This also shows that the interference between carriers depends on the relative frequency deviation and the serial number distance between subcarriers as the relative frequency offset interference factor increases.

When the carrier frequency offset is zero, the interference coefficient is 1. This shows that the interference between carriers depends on the relative frequency deviation and the serial number distance between subcarriers. As the relative frequency offset interference factor increases, *C*(0) reduced interference to the received signal, but *C*(*l* − *k*) increased interference to the received signal; intercarrier interference plays a major role where

(5) $$\begin{array}{c} C\left(l-k\right)=\frac{\text{sin}\left(\pi \left(l+\xi -k\right)\right)}{N\text{sin}\left(\left(\pi /N\right)\left(l+\xi -k\right)\right)}\cdot \exp\left(j\pi \left(\frac{N-1}{N}\right)\left(l+\xi -k\right)\right). \end{array}$$

Equation (5) also shows the energy leakage of subcarrier *k* to subcarrier *l* due to the frequency offset effect. The magnitude of the energy interference depends on the sequence difference of the carrier spacing and the relative carrier frequency offset factor. If the relative carrier frequency offset factor is *e*, then the relative energy leakage of the *k*th carrier to the *l*st carrier can be expressed as

(6) $$\begin{array}{c} {\left|C_{k,l}\right|}^{2}=\frac{{\text{sin}}^{2}\left(\pi \left[\left(l-k\right)+\xi \right]\right)}{N^{2}\cdot {\text{sin}}^{2}\left(\pi \left[\left(l-k\right)+\xi \right]/N\right)}. \end{array}$$

If the interference signal with frequency offset is DFT transformed, it can be written as follows:

(7) $$\begin{array}{c} Y\left(k\right)=X\left(k\right)\cdot C, \end{array}$$

where *C* is the carrier frequency offset interference matrix, which can be expressed as

(8) $$\begin{array}{c} C=\left[\begin{array}{cccc} c\left(0\right) & & \cdots & c\left(N-1\right)\\ c\left(N-1\right) & \ddots & & c\left(N-2\right)\\ \vdots & & c\left(0\right) & \vdots \\ c\left(1\right) & & \cdots & c\left(0\right) \end{array}\right]. \end{array}$$

Equation (8) can obtain some characteristics of the interference matrix *C*. The interference matrix introduced by the frequency offset is a Toeplitz-type matrix, in which each element of the matrix satisfies the periodic cycle property which can be expressed as

(9) $$\begin{array}{c} c\left(k\right)=c\left(k+N\right), \end{array}$$

where (·)^−1^ is the inverse matrix and (·)^*T*^ is the transposed matrix conjugate, which can be expressed as

(10) $$\begin{array}{c} C^{-1}=C^{*}. \end{array}$$

Defining Φ=exp(*j*2*π*Δ*fT*), the elements of the matrix can be written in recursive form:

(11) $$\begin{array}{c} c\left(k\right)=\Phi \cdot c\left(k-1\right)=\Phi^{2}\cdot c\left(k-2\right)=\cdots =\Phi^{i}\cdot c\left(0\right). \end{array}$$

**Figure 2:**
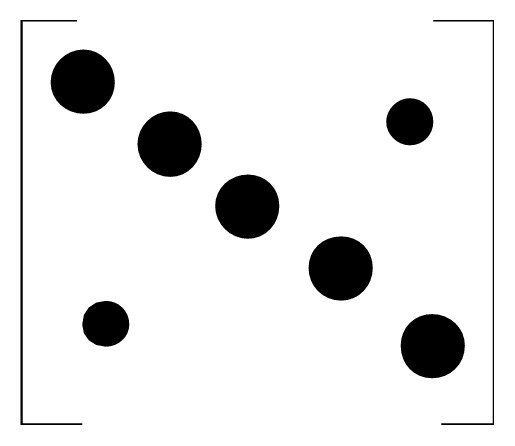
Matrix energy distribution introduced by small frequency offset.

**Figure 3:**
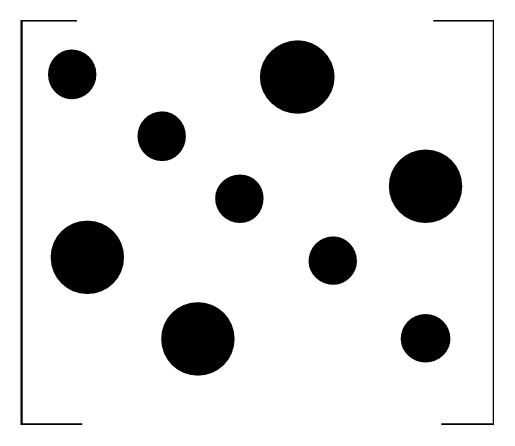
Matrix energy distribution introduced by large frequency offset.

Through the elemental analysis of the matrix, in the case of smaller frequency offset interference, the energy is mainly concentrated on the diagonal. The larger the frequency offset value, the more dispersed the energy, the larger the interference term, and the more the interference of the introduced ICI. Its energy distribution diagram is shown in Figures 2 and 3.

We could obtain from equation (11) that exp(*j*2*πξm*′/*M*) is the linear transformation of the introduced phase.

**Figure 4:**
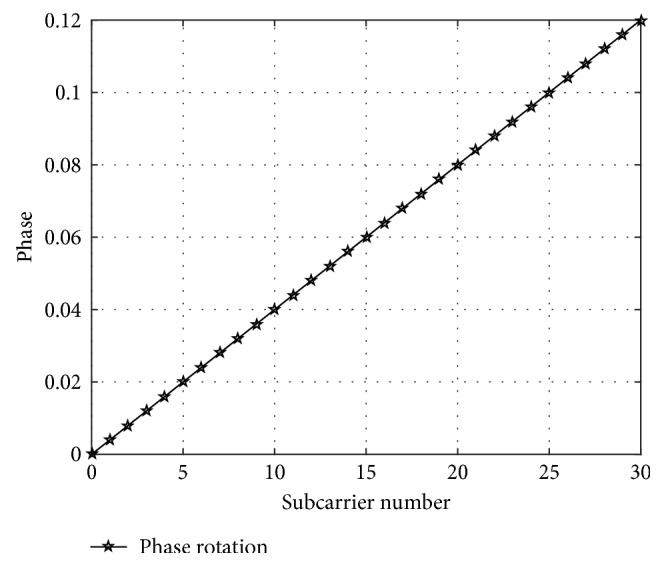
Linear phase introduced by frequency offset.

The number of set carriers is defined as 512, the channel bandwidth is 20 MHz, the Doppler shift is 15 kHz, and the signal mapping mode is QPSK.  Figure 4 shows that the demodulated signal phase changes linearly with the increasing of subcarrier number.

For the multiuser uplink access, the interference comes from frequency offsets. At the same time, the larger the frequency offset range for each user, the more serious the multiuser interference. For the satellite transmission system uplink system, the access interference cancellation of each user is the key for uplink user detection.

## 3. Proposed MUD Algorithm Based on Deep Learning Network

The process of multiuser detection is divided into three parts. First, the multiuser signal is completed to cancel the access interference and the multiuser access interference is reduced by establishing the optimal weight of the multilayer network. Secondly, through the multilayer network, the weight is iterated to obtain the optimal point. Through the network weight sharing and iteration of the first two parts, the optimal identification weight is finally obtained. Multiuser detection and identification is accomplished by optimally identifying the weight network.

### 3.1. Proposed Shared IC Algorithm

The proposed algorithm is based on the goal for optimizing CINR, which is to find the optimal CINR corresponding to WIC interference cancellation algorithm weights. Thus, multiuser interference signal received by satellite can be expressed as

(12) $$\begin{array}{c} Y^{i}\;\;=D^{i}H^{i}C_{i,i}+D^{i}H^{i}C_{i,k}+D^{j}H^{j}C_{i,j}+\;\;N,\;k,i=0,1,\ldots ,\;\;N-1,\;\;j=0,1,\ldots ,M-1, \end{array}$$

where *D*_1_^(*i*)^=[*y*_1_^(*i*)^, *y*_1_^(*i*)^,…, *y*_*M*_^(*i*)^] ∈ *C*^*NxM*^ and *H*^(*i*)^=[*h*_1_^(*i*)^, *h*_1_^(*i*)^,…, *h*_*M*_^(*i*)^]. Here, *M* is the number of subcarrier cancellations and *w* is the weight. *C*_*k*,*l*_ is defined as the interference induced between subcarrier *i* of user *j* and carrier *k* of user *i*, which can be expressed as

(13) $$\begin{array}{c} C_{j,k}=\overset{N-1}{\underset{j=1}{\sum }}\overset{N-1}{\underset{j\neq k^{\prime }}{\sum }}\frac{\text{sin}\pi \left(j-k^{\prime }+\xi^{j}\right)}{N\text{sin}\left(\pi /N\right)\left(j-k^{\prime }+\xi^{j}\right)}\cdot \frac{\exp\left(j\pi \left(j-k^{\prime }+\xi^{j}\right)\right)\left(N-1\right)}{N}. \end{array}$$

According to multicarrier allocation, this process can be a multiuser signal separation. Firstly, each user can be according to the traditional WIC algorithm for cancellation. For user *i*, subcarrier *k*, with the WIC algorithm, it can be expressed as

(14) $$\begin{array}{c} Y_{m}^{i}\left(k\right)=Y_{\left(m-1\right)}^{i}\left(k\right)-w\Lambda_{k,l}Y_{m-1}^{i}\left(l\right)-w\Lambda_{k,l}Y_{m-1}^{j}\left(l\right), \end{array}$$

where *wC*_*k*,*l*_*Y*_*m*−1_^*j*^(*l*) is considered as the previous interference term from subcarrier *l*.

*Y*_*m*_^*i*^(*k*) is defined as the *m*th interference cancellation signals for user *i* and subcarrier *k*, and *Y*_(*m* − 1)_^*i*^(*k*) is the (*m* − 1)th *r* interference cancellation signals for user *j*. Getting formula (14) into the WSIC judgment, it could be obtained as the *m*th interference cancellation signals:

(15) $$\begin{array}{c} Y_{2}^{i}=D^{i}\left(k\right)H\left(k\right)\left(1-wC_{k,l}^{2}\right)+\underset{l\neq k}{\overset{N-1}{\underset{l=0}{\sum }}}\underset{l\in j}{\sum }D^{i}\left(l\right)H^{i}\left(l\right)\left(C_{k,l}\left(1-w\right)-wC_{k,l}^{2}\right). \end{array}$$

Idealizing it, we could obtain

(16) $$\begin{array}{c} {\text{CINR}}^{i}\propto {\left(1-wC_{k,l}^{2}\right)}^{2}. \end{array}$$

Secondly, according to the traditional WPIC algorithm, each user can be for parallel cancellation interference, and it can be expressed as

(17) $$\begin{array}{c} Y_{m}^{i}=Y_{\left(m-1\right)}^{i}-wC_{i,l}Y_{m-1}^{j}, \end{array}$$

where *m* is the iteration for WPIC interference cancellation and *w* is defined as the weight, Θ_*k*,*l*_ is considered as the interference, and *wC*_*i*,*l*_*Y*_*m*−1_^*j*^ is considered as the previous interference term.

*Y*_*m*_^*i*^ is the *m*th interference cancellation signals for user *i* and *Y*_(*m* − 1)_^*i*^ is the (*m* − 1)th interference cancellation signals for user *j*. Getting formula (13) into the WSIC judgment, it could be obtained as the *m*th interference cancellation signals:

(18) $$\begin{array}{c} Y_{2}^{i}=D^{i}\left(k\right)H^{i}\left(k\right)\left(1-wC_{k,l}^{2}\right)+I, \end{array}$$

where *I* is the second interference judgments and *Y*_2_^*i*^ is the received signal after cancellation, which can be obtained as

(19) $$\begin{array}{c} I=\underset{l\neq k}{\overset{N-1}{\underset{l=0}{\sum }}}\underset{l\in j}{\sum }D^{i}\left(l\right)H^{i}\left(l\right)\left(C_{k,l}\left(1-w\right)-wC_{k,l}^{2}\right). \end{array}$$

Due to different user access elevation angles, as well as the satellite ground link fading channel, introducing different serious Doppler frequency shifts is for the satellite-to-ground uplink system. For a particular user, because the tangential velocity of the satellite in a symbol is the same, the relative carrier frequency is kept constant, so the frequency offset introduced in a symbol period can be regarded as constant. Therefore, the normalized frequency offset factor can be considered to be constant.

**Figure 5:**
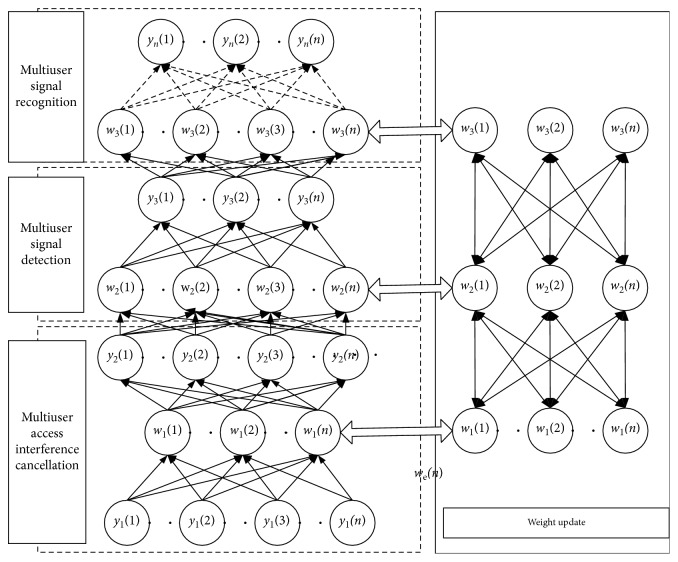
Multiuser detection system block diagram.

The definition of satellite ground link uplink carrier number is 1024; the satellite suburban environment model is given in Table 1.  Figure 5 shows that as the number of carrier interval sequence is reduced, energy leakage is more serious.

It can be obtained from formula (13) that a function of the value of the interference and the *W* weight value with IC algorithm, which can be expressed as a convex function. Signal-to-noise ratio can be obtained to the best value, when the *W* weight is to the pole value. The proposed IC algorithm is based on the optimal weights, which can greatly reduce the number of iterations and improve the accuracy of the algorithm. The signal-to-interference ratio can be used by the comb or block pilot signal. By training the initial weights in the iteration, the optimal interference ratio can be obtained, which is close to the optimum.

**Figure 6:**
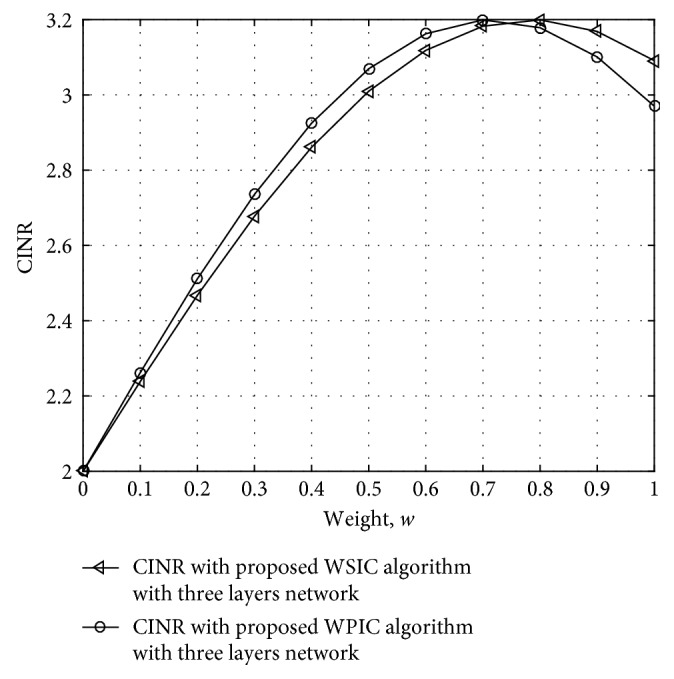
CINR performance at different weights in the IC algorithm (including WSIC and WPIC) with three-layer network in two iterations.

The number of users is 4, and the number of subcarriers is 2048. In the condition of AWGN, SNR = 5 dB, the allocation is OFDM, and the frequency offset is 0.01, 0.05, 0.15, and 0.2. The multifading channel is shown in Table 1. The curve of the relationship between average CINR and weight *w* is shown in Figure 6.

The proposed multiuser detection algorithm is to optimize SINR through obtaining optimal cancellation weight; therefore, the IC algorithm can be divided into WSIC and WPIC algorithms; the WSIC algorithm is cancel interference for multiuser access according to each subcarrier, and the WPIC is the multiuser interference cancellation algorithm at the same time.

Figure 6 states that the convex function can reach the optimal value with *w* weight. Define CINR_opt_ as the optimal after WIC algorithm cancellation. Since CINR_opt_ is the convex function with *w*, the optimal CINR could be obtained when *w* has the extreme. Define the initial value as *w*=1. In this case, the algorithm becomes the traditional SIC or PIC algorithm weight.

The improved algorithm is based on the WIC algorithm. Optimal weights are iterated to approximate initial weights. It is specific for obtaining the optimal weights below.

Defining *E*_CINR_ as the error between CINR_opt_ and CINR_out_, which after cancellation with the WIC algorithm can be expressed as

(20) $$\begin{array}{c} E_{\text{CINR}}={\text{CINR}}_{\text{out}}-{\text{CINR}}_{\text{opt}}, \end{array}$$

CINR_opt_ could be obtained with the Taylor expansion:

(21) $$\begin{array}{c} {\text{CINR}}_{\text{out}}\approx w_{\text{opt}}+\alpha \left(w_{\text{out}}-w_{\text{opt}}\right), \end{array}$$

where

(22) $$\begin{array}{c} \alpha \approx \left[\frac{\partial {\text{CINR}}_{\text{out}}}{\partial w_{\text{out}1}},\frac{\partial {\text{CINR}}_{\text{out}}}{\partial w_{\text{out}2}},\ldots ,\frac{\partial {\text{CINR}}_{\text{out}}}{\partial w_{\text{out}k}}\right]. \end{array}$$

The influence of the satellite to ground link on this algorithm consists of two parts. Firstly, the influence for the proposed algorithm is also induced by the multiuser access angle differences. Due to differences in relative motion between the user and satellite, multiuser access interference has been generated, which significantly degrades the satellite system performance.

When multiusers access the same satellites, the multiuser access angle differences will introduce different carrier frequency offsets in the total number of the carrier system. Under certain conditions, the carrier frequency deviation will induce the different multiple access interference, including interference simulation as shown in Figure 6; the number of users is increasing, and the serious interference induced by frequency offset is larger.

Secondly, the influence for the proposed algorithm is induced by multipath fading. For urban simulation scenarios, the signal reflection effect caused by buildings is larger, and the diffraction effect caused by multipath is also larger. The more delay the received signal propagation, the more serious the signal fading is.

In the countryside scene, compared with the urban scene, the multipath number is decreased and the fading is relatively flat. This is because that the contryside scene is with a smaller number of building and weaker reflection and refraction.

### 3.2. Shared Weight Process and Feedback Solution Process

For multiuser received signals to cancel interference, we use shared weights to obtain the best weights and then obtain user detection and weight update.

The cost function established can be expressed as

(23) $$\begin{array}{c} \underset{x}{\text{min}}\lambda_{t}{\left\|w_{t}\right\|}_{1}+{\left\|y_{t}-w_{t}x\right\|}_{2}^{2}. \end{array}$$

The cost function is to find that the accurate reconstruction, which should be realized. Then, the optimal weight detection error is made. Therefore, the sparse recovery for multiweight sharing can be obtained, and the optimal user detection for all the users can be satisfied in the following:

(24) $$\begin{array}{c} \underset{x}{\text{min}}\lambda_{t}{\left\|w_{t}\right\|}_{1,w}+{\left\|y_{t}-w_{t}x_{t}\right\|}_{2}^{2}, \end{array}$$

where

(25) $$\begin{array}{c} {\left\|w\right\|}_{1,w}=\overset{K}{\underset{i=1}{\sum }}w_{i}\left|x_{i}\right|. \end{array}$$

The first term is a nonzero regular term whose position is known and is different from the traditional mode of all cost functions.

In addition to the weight constraint cost, the regularization constraint can be established for the corresponding *x*.

Therefore, when the measurement data are very small, *x* would become larger. For solving the problem, we used the improved solutions to solve the regularization. The modified cost function is expressed as

(26) $$\begin{array}{c} \underset{x}{\text{min}}\lambda_{t}{\left\|w\right\|}_{1,w}+\alpha_{t}{\left\|x-{\hat{x}}_{t}\right\|}_{2}^{2}+{\left\|y_{t}-w_{t}x\right\|}_{2}^{2}. \end{array}$$

The augmented Lagrangian is expressed as

(27) $$\begin{array}{c} L\left(x,z,t_{t}\right)=\alpha_{t}{\left\|x-{\hat{x}}_{t}\right\|}_{2}^{2}+{\left\|y_{t}-w_{t}x\right\|}_{2}^{2}+t_{t}{\left\|w-{\hat{w}}_{t}\right\|}_{2}^{2}. \end{array}$$

The scaled problem (6) consists of three iterations:

Shared weight updates: *x*, *w*, and *u*.

(28) $$\begin{array}{c} x_{t}^{\left[k+1\right]}=\underset{x}{\arg\text{min}}\left(\alpha_{t}{\left\|x-{\hat{x}}_{t}\right\|}_{2}^{2}+{\left\|y_{t}-w_{t}x\right\|}_{2}^{2}+\beta {\left\|w-w_{t}^{\left[k\right]}+u_{t}^{\left[k\right]}\right\|}_{2}^{2}\right),\\ w_{t}^{\left[k+1\right]}=\underset{w}{\arg\text{min}}\left({\left\|w-{\hat{w}}_{t}\right\|}_{2}^{2}+\beta {\left\|w_{t}^{\left[k+1\right]}-w_{t}^{\left[k\right]}+u_{t}^{\left[k\right]}\right\|}_{2}^{2}\right),\\ u_{t}^{\left[k+1\right]}=u_{t}^{\left[k+1\right]}+x_{t}^{\left[k+1\right]}-w_{t}^{\left[k+1\right]}. \end{array}$$

## 4. Experimental Classification Results and Analysis

Establishing that the orbital altitude is 1100 km, the rural environment measured data proposed in [36] are satellite-to-ground uplink system model as in Table 2 in this paper.

Set the satellite beam spot beam of number 5 with a coverage diameter of 450 km. Satellite-to-ground link model with *L*-band carrier frequency in rural environments is shown in Table 2. Set the number of uplink users 4 and transmit pilot block type information. For analysis, the user has access to a maximum height at 35°, 25°, 5°, and 15°. The signal bandwidth is 50 MHz, the number of subcarriers is 1024, and the signal mapping method is defined as QPSK, BPSK, 16QAM, OFDM signal.

**Figure 7:**
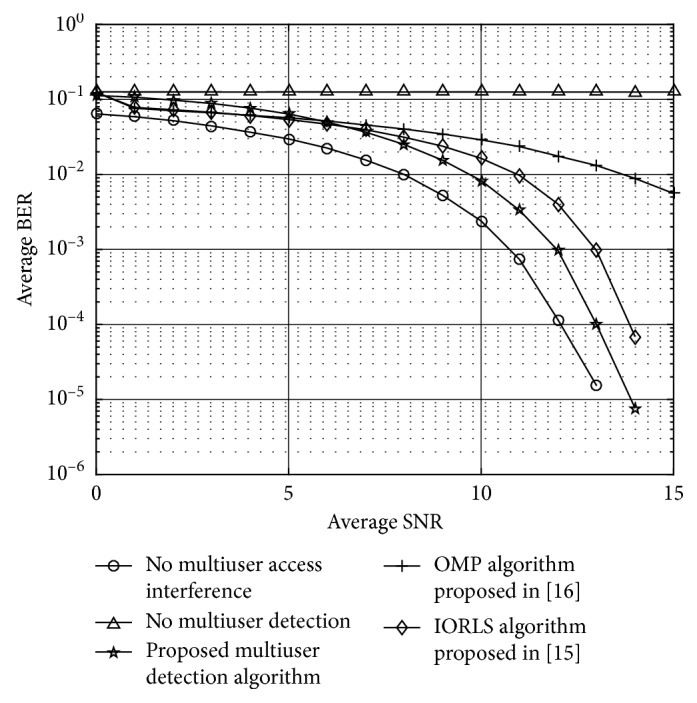
BER curves under different SNRs.

Figure 7 shows the BER simulation results for multiuser detection based on the WIC algorithm. Compared with the iterative sorting least squares (IORLS) algorithm proposed in [12] and the orthogonal signal tracking (OMP) algorithm proposed in [13], the WIC algorithm is improved by two iterations for sharing weight. The simulation results can be obtained from Figure 7. In first iteration, the multiuser detection algorithm performs better than the WPIC algorithm. Through the optimization iteration, the weight converges to the bump function close to the inflection point, and we can obtain the optimal multiuser detection and thus obtain the best BER performance for multiuser detection. Since the proposed MUD algorithm is superior to the improved conventional algorithm, interference cancellation improves the complexity. The system is enhanced under the condition of 10*e* − 3 BER.

**Figure 8:**
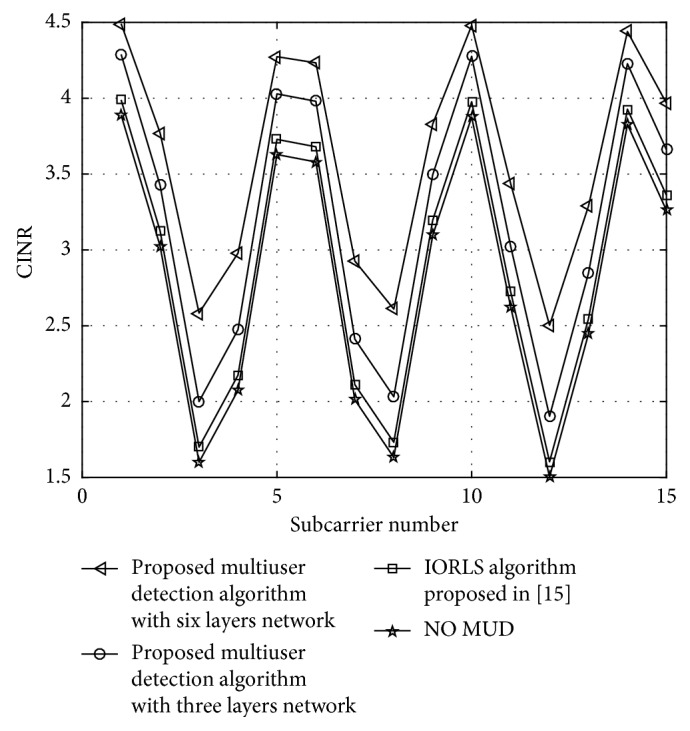
CINR performance of different subcarriers.

Figure 8 shows the CINR performance comparison between the MUD algorithm proposed in [12] and the proposed MUD algorithm, since the improved MUD algorithm cancels subcarrier frequency offset interference and CINR system can be optimal.

**Figure 9:**
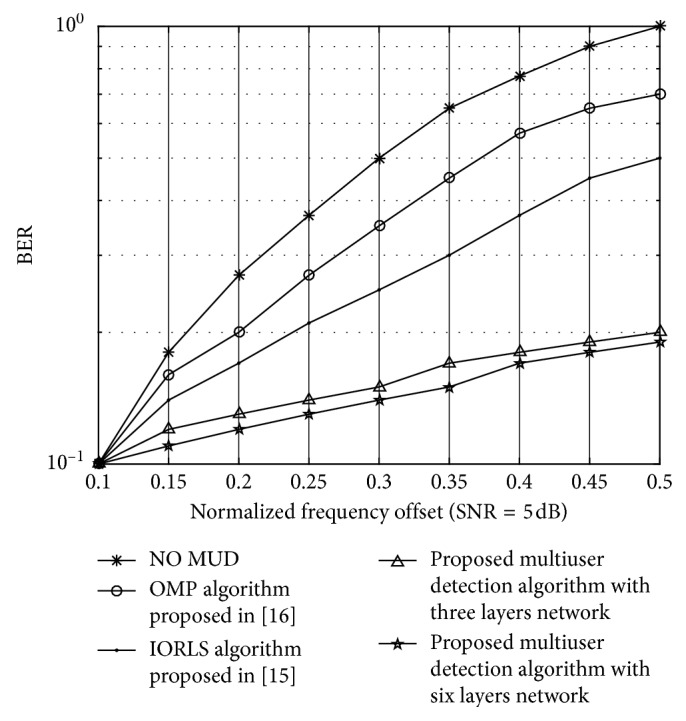
BER performance of different normalized frequency offsets.

Figure 9 shows different BER curves for each uplink user at SNR = 5 dB. As the relative interference carrier frequency offset increases, the BER of user error rate increases, which is due to the multiuser access system. And its performance of the algorithm is similar to the traditional algorithm, which is due to interference introduced by carrier frequency offset. After multilayer iteration, the weight is close to the optimal value at this time, and then the system is close to the maximum SINR optimization, which can be obtained as an optimization.

**Figure 10:**
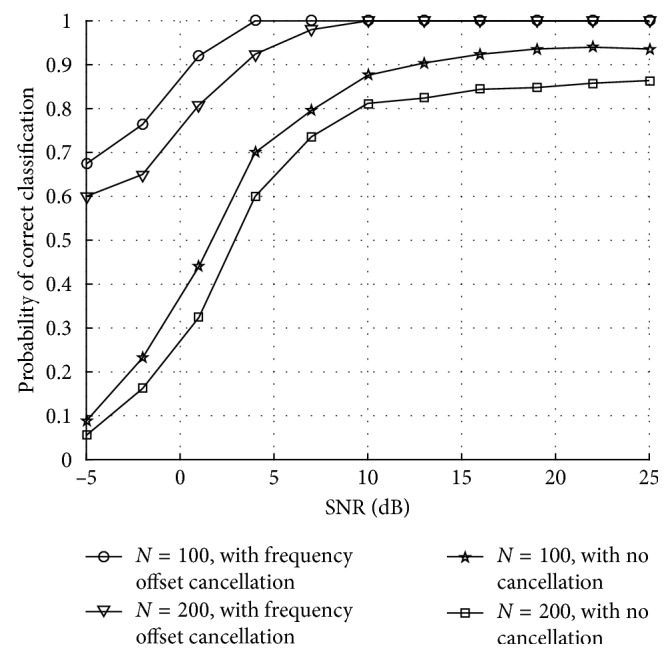
Probability of correct classification of the proposed deep learning network for multiuser access to satellite at different length curves.

Figure 10 is the probability of correct classification of proposed deep learning network at different SNRs, which is also based on different length curves for each user. As the relative interference carrier frequency offset increases, the PCC of user error rate increases, which is due to the user access to the multiuser access system. The performance of the improved traditional multiuser detection algorithm at large freuqency offset is poor, which is due to residual carrier frequency offset. When training length is larger, the system is close to the maximum SINR optimization, which can be obtained as an optimization.

## 5. Conclusion

A multiuser detection algorithm based on deep learning network has been proposed. The proposed deep learning network for MUD could provide high precision and lower iteration times, which firstly establishes the CINR optimal loss function according to the multiuser access interference mode and then obtains the best multiuser detection weight through the steepest gradient iteration. The important feature of the proposed algorithm is through nonlinear optimal direction learning and to achieve maximum signal-to-noise ratio through gradient iteration, and then share weights. Through establishing a typical satellite communication system simulation platform, compared with the OMP and IORLS algorithms, the proposed deep learning network algorithm has better performance in different conditions of SNR, CINR, and carrier frequency offset interference.

## Figures and Tables

**Table 1:** Suburban environment parameter.

Tap	Distribution function	Parameter	Parameter distribution	Numerical value (dB)	Time delay (ns)
1	LOS : Rician	Rice factor	*K*	9.7	0
2	Rayleigh	Average multipath power	2*σ*_*l*_^2^	−23.6	100

**Table 2:** Channel model parameter in rural environment.

Tap	Distribution function	Parameter	Parameter distribution	Numerical value (dB)	Time delay (ns)
1	LOS : Rician	Rice factor	*K*	6.3	0
	nLOS : Rayleigh	Average multipath power	2*σ*_*l*_^2^	−9.5	
2	Rayleigh	Average multipath power	2*σ*_*l*_^2^	−24.1	100
3	Rayleigh	Average multipath power	2*σ*_*l*_^2^	−25.2	250

## Data Availability

The data used to support the findings of this study are available from the corresponding author upon request.
